# Lost in the Haystack: Smaller Needles are More Difficult for LLMs to Find

**Published:** 2025-05-23

**Authors:** Owen Bianchi, Mathew J. Koretsky, Maya Willey, Chelsea X. Alvarado, Tanay Nayak, Adi Asija, Nicole Kuznetsov, Mike A. Nalls, Faraz Faghri, Daniel Khashabi

**Affiliations:** 1 Center for Alzheimer’s Disease and Related Dementias, NIA, NIH; 2 DataTecnica LLC; 3 Johns Hopkins University; 4 Laboratory of Neurogenetics, NIA, NIH

## Abstract

Large language models (LLMs) face significant challenges with needle-in-a-haystack tasks, where relevant information (“the needle“) must be drawn from a large pool of irrelevant context (“the haystack“). Previous studies have highlighted positional bias and distractor quantity as critical factors affecting model performance, yet the influence of *gold context size* has received little attention. We address this gap by systematically studying how variations in gold context length impact LLM performance on long-context question answering tasks. Our experiments reveal that LLM performance drops sharply when the gold context is shorter, i.e., **smaller gold contexts consistently degrade model performance and amplify positional sensitivity,** posing a major challenge for agentic systems that must integrate scattered, fine-grained information of *varying lengths*. This pattern holds across three diverse domains (general knowledge, biomedical reasoning, and mathematical reasoning) and seven state-of-the-art LLMs of various sizes and architectures. Our work provides clear insights to guide the design of robust, context-aware LLM-driven systems.

## 1 Introduction

Large language models (LLMs) increasingly power applications that require reasoning over vast amounts of information, ranging from synthesizing findings across scientific literature [1–7], to navigating complex codebases [8–10], to maintaining coherence in multi-turn conversations. These applications share a common requirement: strong long-context understanding. This is particularly vital for *agentic systems*, in which autonomous agents must integrate heterogeneous information streams from specialized components to reason, plan, and act effectively.

A critical stage in such systems is *aggregation*, the synthesis of retrieved evidence into an accurate, actionable response. This stage determines what content to include, cite, or ignore, and has direct implications for safety, reliability, and factual correctness. Aggregation becomes especially challenging in *needle-in-a-haystack* scenarios, where relevant evidence (the ‘gold context’) is embedded within a large volume of topically related or superficially plausible but ultimately irrelevant or misleading, ‘distractor context’ [11, 12]. Successful aggregation requires precise identification and prioritization of minimal but essential content while discarding noisy signals.

Although LLMs now support context windows stretching into the millions of tokens, recent studies show that simply increasing input length does not ensure strong long-context reasoning. Prior work has explored *positional bias* [13–15], showing that early content is more likely to be attended to, and that distractors degrade performance. However, one key dimension remains underexplored: *how the size of the gold context influences model performance?*

In this study, we systematically examine how gold context size affects long-context performance in LLMs. Specifically, we adapt three diverse benchmarks, CARDBiomedBench [16] (biomedical reasoning), NaturalQuestions [17] (general knowledge), and NuminaMath1.5 [18] (mathematical reasoning), to vary both the size and position of the gold context while keeping the distractor content fixed (see Section 2).

Our key findings are:

1. Smaller gold contexts lead to significantly worse performance.
2. Smaller gold contexts exhibit higher positional sensitivity.

Models are remarkably more sensitive to the placement of smaller gold spans, with accuracy declining when relevant content appears later in the context window. This effect holds across seven state-of-the-art open and closed-weight LLMs spanning diverse architectures and scales. Notably, models achieve near-perfect scores in no-distractor settings, confirming that these failures are due to aggregation breakdowns rather than task difficulty (see Section 3).

Further exploration provides additional insights into the nuances of aggregation (Section 4). We observe a pronounced primacy bias for smaller gold contexts, wherein relevant information placed near the beginning of the context window is more reliably utilized by models. This effect diminishes with larger gold contexts, which are more resilient to positional variation. The problem is amplified in domain-specific reasoning (e.g., biomedical and mathematical tasks), and exacerbated as distractor size increases. We also find that domain-specific tasks exacerbate the challenges posed by small gold contexts relative to general-knowledge tasks. Last but not least, we observe that this trend persists as number of distractor documents increases.

These findings carry practical implications. In real-world deployments, context size, position, distractor size, and noise are rarely controllable. Aggregation failures due to overlooked gold context size can degrade trust, safety, and downstream task performance. Based on our empirical findings, we offer guidelines for designing more robust aggregation strategies for agentic systems, including techniques for structuring and expanding critical evidence to reduce fragility.

In sum, we show that the size of relevant evidence—*not just its location*—is a critical factor in long-context reasoning. Our results highlight an often-overlooked bottleneck in LLM capabilities and offer both diagnostic insights and prescriptive tools for practitioners building context-resilient AI systems.

## 2 Experimental Setup

We designed our experiments to systematically evaluate how the size of the gold context affects long-context LLM performance. This section outlines our core design objectives, benchmark adaptations, baseline validations, and primary evaluations simulating realistic aggregation settings.

### 2.1 Desiderata

To systematically evaluate the impact of gold context size on long-context LLM aggregation performance, our guiding desiderata were (1) **Realism**, (2) **Gold Size Variability**, (3) **Distractors**, and (4) **Generality**.

#### Realism.

In real-world agentic systems, aggregation typically involves synthesizing outputs from multiple specialized agents, each retrieving documents from their domain of expertise. Usually, one agent returns the document containing the correct answer (the “gold” document), while others provide distractors, topically relevant but ultimately uninformative documents. We simulate this by inserting a gold document of varying size at different positions within a fixed-length sequence of distractors. The document order is randomized to reflect natural uncertainty in agent contributions and retrieval quality.

#### Gold Size Variability.

To isolate the impact of gold context size, we constructed three nested variants for each benchmark:

- **Small Gold**: Minimal span sufficient to answer the question.
- **Medium Gold**: Additional explanatory or supporting content.
- **Large Gold**: Complete reasoning process and/or extended relevant context.

These were wrapped as pseudo-documents, including metadata such as titles and questions when appropriate. All variants were hierarchically structured (small ⊂ medium ⊂ large) and validated for sufficiency. See 2 for examples. Performance is very high and uniform when observing solely any size of gold as can be seen in Table 1.

#### Distractors.

To simulate realistic confounding signals, we curated distractors that are topically relevant and lexically similar to the question but do not contain the answer. Distractor quantities per benchmark were calibrated to match token distributions observed in a real-world multi-agent retrieval system (∼ 20*k* tokens).

#### Generality.

We selected three diverse benchmarks spanning biomedical, general knowledge, and mathematical reasoning, and evaluated performance across seven leading LLMs of varying architecture and scale. This ensures that our findings generalize across domains and model classes.

### 2.2 Task Construction: Needles and Haystacks

**Figure 3:**
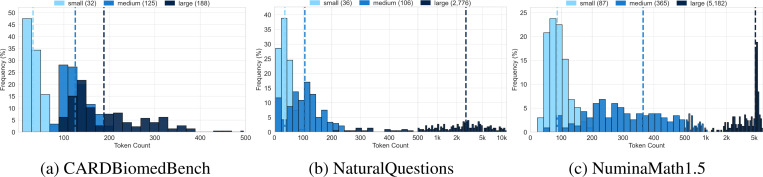
Token count distributions for the varying sizes of gold context on each benchmark. Median token count is in parenthesis in the legend. X-axis is dynamically scaled as linear (0–500 tokens) and logarithmic (500+ tokens) for visibility.

We adapted three established question and answering benchmarks—CARDBiomedBench (biomedical reasoning), NaturalQuestions (general knowledge), and NuminaMath1.5 (mathematical reasoning)—to create controlled needle-in-a-haystack settings. Gold context sizes were varied, accompanied by distractors explicitly designed to be topically relevant yet answer-free. Figure 3 displays token count distributions for the varying sizes of gold.

#### CARDBiomedBench (CBB).

CBB is a question-answering dataset focused on neurodegenerative diseases (NDDs), designed to evaluate LLM performance on biomedical reasoning tasks involving genetic, molecular, and clinical information. The BiomedSQL [19] variant augments each example with SQL queries and database rows to support structured reasoning experiments. This provides the following sizes of gold:

*Small*: Answer only. *Medium*: SQL query + answer. *Large*: SQL query + returned rows + answer.

Distractors were sampled from documents retrieved by four independent domain-specific agents in a real-world system. These documents are semantically relevant but verifiably do not contain the answer, presenting realistic aggregation challenges.

#### NaturalQuestions (NQ).

NQ is an open-domain QA benchmark using real Google user queries, with evidence-linked Wikipedia passages from the KILT corpus [20]. Gold contexts were derived as:

*Small*: Sentence containing the answer. *Medium*: Full paragraph around the sentence. *Large*: Paragraph ± 4 adjacent paragraphs.

Distractors were drawn from the HELMET [21] adaptation of NQ-KILT, using dense retrieval over 2019 Wikipedia with the gte-large-en-v1.5 embedding model [22]. From HELMET’s 100-token chunks, we excluded documents: (1) explicitly labeled as gold evidence and (2) containing the answer string to ensure high semantic similarity without answer leakage. This ensured the distractors are lexically and topically aligned with the question, but verifiably devoid of answer content.

#### NuminaMath1.5 (NM).

NM is the largest open-source dataset for math reasoning, with problems ranging from high school to International Mathematical Olympiad (IMO)-level difficulty, originating from diverse sources like Chinese K-12 exams, AMC/AIME contests, and global math forums. We used the OpenR1Math [23] variant, which includes model-generated solution traces from DeepSeekR1 [24] verified for correctness. We filtered for examples with complete reasoning streams and defined gold variants as:

*Small*: Final answer. *Medium*: Textbook-style solution + answer. *Large*: Full LLM-generated chain-of-thought + solution + answer.

Distractors were unrelated reasoning traces matched for length and complexity. Due to length variability, large gold contexts were capped at the final 5k tokens, which included concluding reasoning and answers.

### 2.3 Baseline Experiments

To validate our construction, we ran three baseline conditions:

#### Closed-book.

No context was provided, assessing whether models could answer from internal knowledge. This gauges possible benchmark saturation.

#### Gold-only.

Each gold context size (small, medium, large) was presented alone, without distractors. This verified that all variants were independently sufficient to solve the task and that downstream performance drops are due to aggregation effects (e.g., distractor interference or gold placement). Table 1 contains a subset of such baselines.

#### Distractor-only.

Models were given only distractor documents. For CBB, we also tested distractors from each agent separately to confirm they were individually non-informative. These checks ensured that distractors lacked sufficient signal to answer correctly.

These baselines serve to validate that observed performance differences in main experiments result from changes to gold size, rather than underlying flaws in dataset or distractor construction.

### 2.4 Main Experiments

We simulated realistic aggregation scenarios by embedding each gold context size at varying positions within a fixed sequence of distractors. This tests both gold size sensitivity and positional sensitivity simultaneously.

We evaluated seven leading LLMs:

- **Closed-weight:** GPT-4o [25], GPT-4o-Mini [26], Gemini-2.0-Flash, Gemini-2.0-Flash-Lite [27]
- **Open-weight:** LLaMA-3.1–405B, LLaMA-3.3–70B, LLaMA-3.1–8B [28]

Each model was evaluated on every size-position combination in a controlled, deterministic setting (temperature = 0). Prompts were standardized within each benchmark. This setup enables rigorous, cross-model evaluation of gold context sensitivity and simulates the type of unpredictable document ordering common in deployed LLM systems.

## 3 Empirical Findings

Our experiments reveal that the size of the gold context has a substantial and consistent effect on long-context performance across benchmarks, models, and domains.

### 3.1 Smaller Gold Contexts Lead to Lower Performance

**Figure 4:**
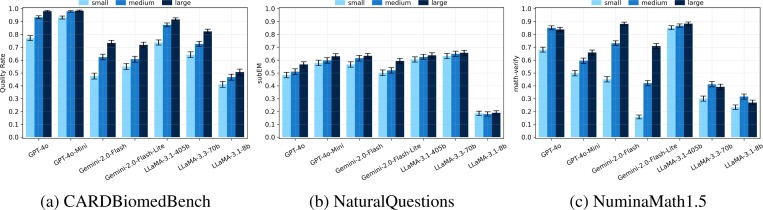
Average performance across all gold positions for each benchmark and gold context size. Metrics are benchmark-specific (BioScore, subEM, math-verify). Higher is better. Error bars indicate 90% confidence intervals. Colors correspond to gold context sizes: small, medium, large. **Across all settings, performance improves monotonically with gold context size.**

Across all benchmarks and models, increasing the size of the gold context significantly improves accuracy (Figure 4). For example, on CARDBiomedBench (CBB), Gemini-2.0-Flash improved from 48% with small gold to 62% with medium and 73% with large gold contexts. GPT-4o showed a similar trend, rising from 77% (small) to 98% (large), while LLaMA-3.1–405B improved from 74% to 92%.

Notably, performance with large gold contexts closely approached the model-specific *gold-only baselines* (i.e., accuracy when the gold context is shown without any distractors) recorded at 96% for Gemini-2.0-Flash, and 100% for both GPT-4o and LLaMA-3.1–405B. This suggests that large gold contexts allow models to nearly recover ideal aggregation performance, while small golds fall significantly short.

### 3.2 Smaller Gold Contexts Are More Sensitive to Position

**Figure 5:**
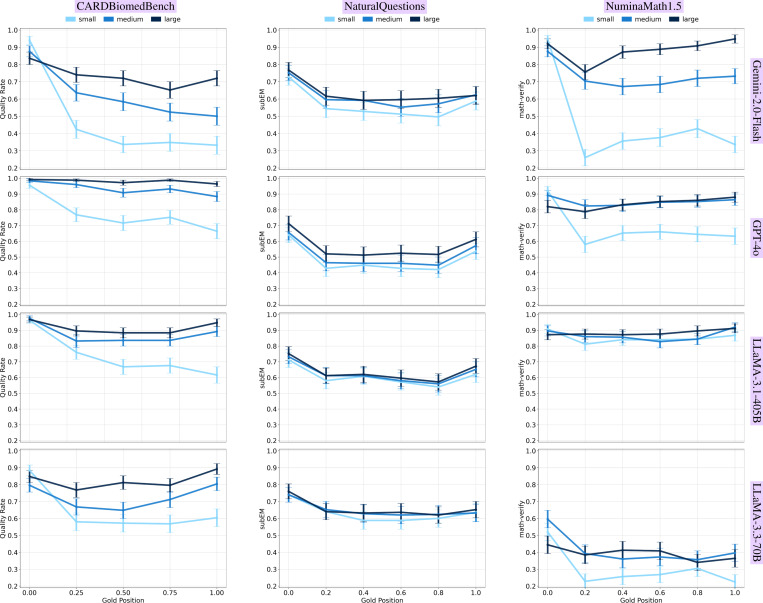
Model performance by gold context position (early to late in input), higher is better and error bars are 90% CIs. Each row is a model, columns are benchmarks. **Smaller gold contexts exhibit sharper performance degradation with later placement, especially in specialized domains (CBB, NM).** Larger contexts mitigate this sensitivity, highlighting the stabilizing effect of richer input.

We observed a clear positional sensitivity effect that was amplified in smaller gold contexts (Figure 5). Performance systematically declined when small gold contexts appeared later in the input, while large gold contexts were more robust to position.

For instance, in CBB, Gemini-2.0-Flash achieved 94% accuracy when the small gold context was placed at the start of the context window, but only 33% when placed near the end, a 61-point drop. In contrast, the large gold context declined more gradually, from 84% to 65%, demonstrating greater positional resilience. This pattern held across all evaluated models and benchmarks.

Importantly, the positional effect was more pronounced in domain-specific tasks (e.g., biomedical and math) than in general knowledge (NQ), suggesting that both information type and gold size compound aggregation difficulty.

These empirical results establish that both gold context size and position critically affect long-context reasoning. In the next section, we delve deeper into these patterns, analyzing where, why, and how these failures manifest at finer granularity.

## 4 Additional Analysis

Beyond our core findings, we conduct further analyses to understand *why* smaller gold contexts lead to performance degradation, and under *which conditions* these effects are most severe. Specifically, we examine the impact of domain specificity, positional variance, and distractor volume.

### Domain-Specific Tasks Amplify Sensitivity to Gold Context Size.

**Figure 6:**
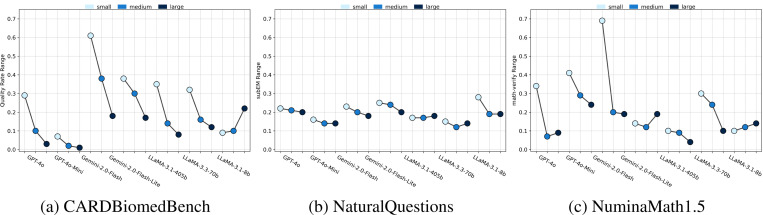
Positional sensitivity by benchmark. For each model and gold context size, we compute the range (Equation 1) of performance across positions. **Smaller gold contexts exhibit much higher sensitivity (larger ranges), especially in domain-specific tasks (CBB, NM). Larger gold contexts yield more stable performance across positions.**

The effects of gold context size are notably amplified in domain-specific tasks compared to general knowledge. Figure 6 quantifies this by measuring the range in model performance across different gold context positions. For each model and gold size, we compute the performance range as the difference between maximum and minimum scores across all positions:

(1) $$\text{Range}=\underset{i\in \{1,\ldots ,n\}}{\max}\text{perf}\left({\text{position}}_{i}\right)-\underset{i\in \{1,\ldots ,n\}}{\min}\text{perf}\left({\text{position}}_{i}\right)$$

For example, on NuminaMath1.5, Gemini-2.0-Flash showed a performance range of 72% for small gold contexts, compared to only 20% for large gold. A similar pattern held in CARDBiomedBench. In contrast, NaturalQuestions exhibited smaller variation across all sizes, likely due to easier questions and higher closed-book baseline scores. This suggests that general knowledge tasks may be inherently more resilient to gold context variability.

### Smaller Gold Contexts Exhibit Stronger Primacy Bias.

We also observed a primacy bias across models, performance was consistently higher when the gold context appeared early in the input window. This effect was especially pronounced for small gold contexts. In some cases, small gold contexts placed at the beginning of the input even outperformed medium or large contexts placed later, despite their reduced information content. This occurs often in the left and right columns of Figure 5, where the small gold line starts at the top at gold position 0.0 before crossing over to the bottom.

This inversion highlights the sensitivity of model attention to positional cues when dealing with minimal evidence. While some bias exists for larger contexts, they are substantially more robust to position and do not exhibit the same sharp drop in middle and tail placements.

### Gold Context Size Remains Critical as Distractor Volume Increases.

To evaluate the robustness of the gold context size effect under varying degrees of context noise, we systematically increased the number of distractor documents. We leveraged our flexible adaptation of NuminaMath1.5 to run experiments with 5, 10, and 15 distractors, approximately 25k, 50k, and 75k distractor tokens, respectively.

**Figure 7:**
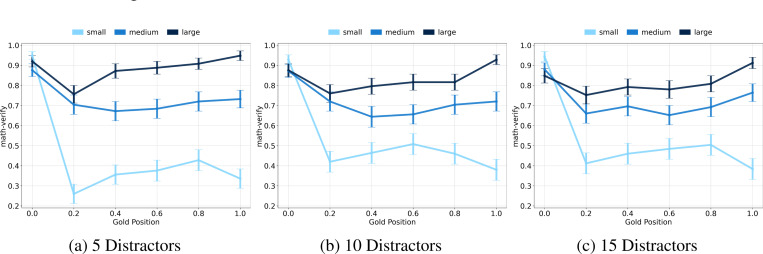
Gemini-2.0-Flash performance on NuminaMath1.5 as the number of distractor documents increases (error bars are 90% CIs). Despite growing distractor noise (up to ∼75k tokens), the performance gap between small and large gold contexts persists. **This confirms that gold context size remains a key factor in long-context reasoning under high-noise conditions.**

Figure 7 shows that performance continues to be strongly influenced by gold context size, regardless of distractor volume. This reinforces that gold context size remains a dominant variable, even when noise levels change.

### Summary.

These additional analyses confirm that the observed effects are not artifacts of a single benchmark or setup. Small gold contexts not only reduce performance, but also magnify positional bias. These effects are more severe in noisy environments and domain-specific tasks.

## 5 Related Work

We review related work in the context of long-context reasoning, focusing on three themes: positional biases in LLMs, long-context evaluation frameworks, and mitigation strategies.

### Positional biases in LLMs.

Position bias, the tendency of LLMs to over- or under-attend to different parts of the input, has emerged as a fundamental challenge. Prior work has identified several variants: *primacy bias*, where early content is favored [13]; *recency bias*, where later content dominates [15]; and *U-shaped bias*, where mid-context is under-attended [14]. These effects persist across model architectures, alignment strategies [14], extended context lengths [29], and even, to some extent, in internal representations [30]. Our work contributes to this literature by introducing a new dimension: we show that *the size of the gold context modulates the strength of positional bias*. Specifically, smaller gold contexts are significantly more vulnerable to primacy effects, while larger contexts confer greater robustness to positional variation.

The closest work to our setup is recent work of Levy et al. [31] who study needle-in-a-haystack performance under variable input lengths. While both works investigate positional dynamics in noisy settings, our approach holds the distractor context fixed and instead varies the gold context size, allowing us to isolate the effects of gold signal sparsity.

### Frameworks for long-context evaluation.

Evaluation strategies for long-context reasoning have evolved from synthetic toy tasks to richer, more realistic setups. Long-Range Arena [11] introduced standardized tasks for comparing various transformer variants. Recent benchmarks explore broader benchmarking variations [32–48], such as document synthesis [12, 49], document-level retrieval [21], citation verification [50], and biomedical reasoning [6, 51]. Most of these setups use the “needle-in-a-haystack” formulation [52, 53] where a small relevant span must be retrieved from a large set of distractors. Some efforts push beyond this setup, incorporating aggregation, multi-hop inference [54, 55], or mixed-modality inputs [56]. Our work builds on this direction by adapting natural, domain-specific datasets to simulate realistic multi-agent aggregation within a ‘needle-in-a-haystack” framework due to its practical relevance.

### Mitigation strategies for position bias.

Several mitigation approaches have been proposed to reduce position sensitivity in LLMs. These include compressing or abstracting context [57], distilling long-context information into weights [58], reweighting attention via calibration [59], modifying positional encoding schemes [60, 61], and fine-tuning on debiased distributions [62]. While some methods mitigate positional biases, many introduce side effects [63], leaving long-context generalization an ongoing challenge. Our contribution is diagnostic rather than corrective. We uncover a novel interaction between input structure (gold context size) and positional bias severity, showing that simply increasing the amount of gold evidence can systemically impact position bias. Whether existing mitigation strategies can address this effect remains an open question for future work.

## 6 Discussion, Limitations, and Conclusion

### Why does gold context size strongly affect aggregation accuracy?

Our findings reveal two interconnected factors: First, we hypothesize that larger gold contexts attract attention by offering a *higher density* of semantically relevant tokens, making them more prominent within distracting content. This richer semantic environment helps models retrieve relevant signals and reduces positional sensitivity. The effect is especially pronounced in domain-specific tasks, where coherent reasoning chains in larger contexts help models follow structured logic needed for accurate answers.

### Practical implications of our findings.

While prior work has studied factors like positional bias and distractor count, we highlight an overlooked and less controllable factor: gold context size. Therefore, practitioners should recognize that aggregation quality is sensitive to context length variations, even when retrieval mechanisms functions as expected. Practitioners can address this by strategically balancing retrieved document sizes and accounting for potential biases against shorter contexts.

### Limitations of our study.

First, we did not explicitly control the proportion of gold context within the total context window. Instead, we fixed distractor lengths to better reflect real-world conditions, resulting in varying gold-to-distractor ratios. This may confound whether performance differences stem from gold context size alone or its relative share. Second, while our benchmarks and distractors were curated for realism and domain diversity, only the CBB dataset used a real-world retriever; NQ and NM relied on synthetic setups. Future work should address these.

### Conclusion.

Our study reveals a fundamental yet previously overlooked limitation in LLM aggregation capabilities: **the size of relevant information critically influences aggregation effectiveness in long-context tasks.** Through systematic evaluation, we demonstrated that smaller gold contexts degrade model performance substantially and exacerbate positional sensitivity, especially in domain-specific tasks. This discovery underscores a crucial vulnerability in real-world agentic deployments, where relevant evidence often appears unpredictably scattered amidst extensive distractors. As language models become central to applications requiring precise and trustworthy reasoning-from scientific discovery to personalized assistants-our findings highlight the urgent need to rethink aggregation strategies. Future LLM-driven systems must explicitly address context-size variability to ensure reliability, safety, and user trust in the face of complex, noisy real-world information streams.

## Figures and Tables

**Figure 1:**
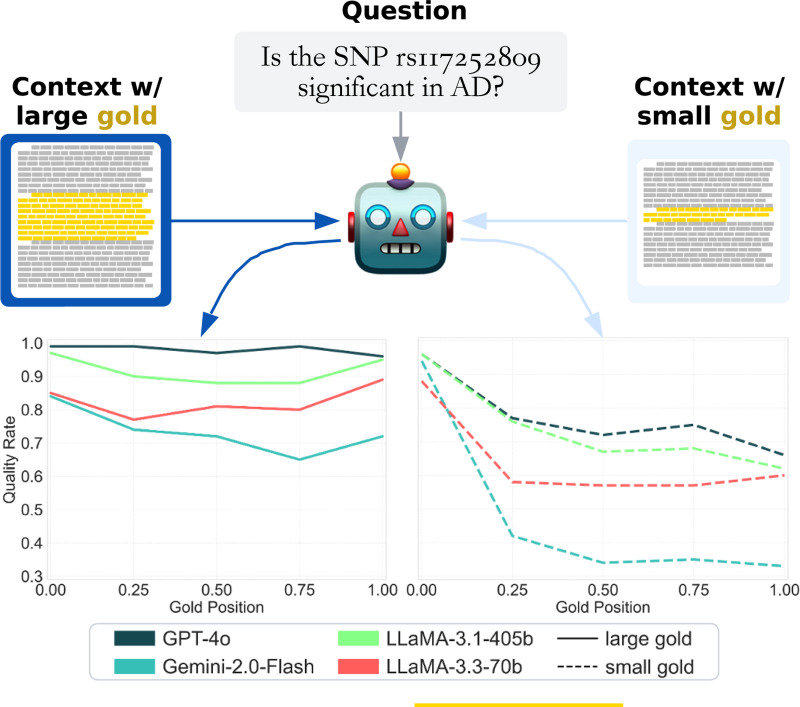
Changing both the size and position of gold context (relevant information) within a set of distracting context(irrelevant information), we observe that LLM Needle-In-A-Haystack Performance is *overall lower* and *more sensitive to position* when models are given short gold documents (dashed line) as opposed to long (solid line).

**Figure 2:**
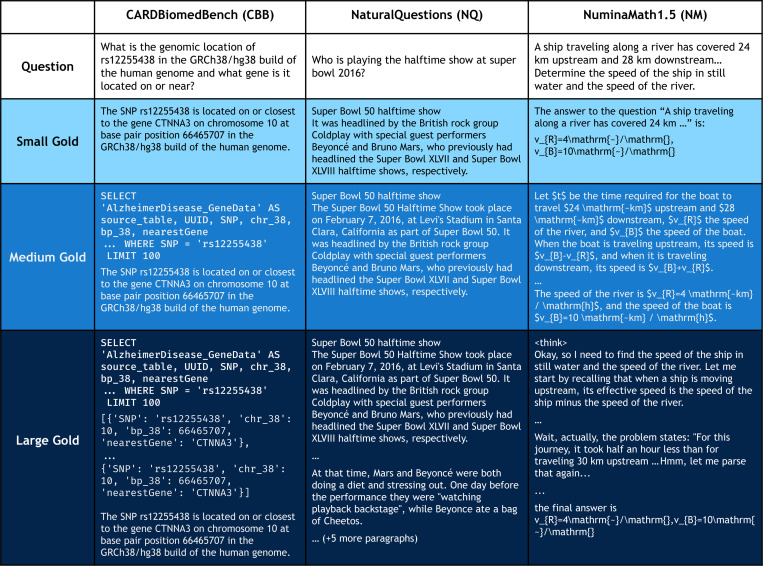
Gold context construction across benchmarks. The “small” gold context is minimally sufficient to answer the question; “medium” and “large” add further relevant information. In CARDBiomedBench (left), this includes SQL and result rows; in NQ (center), adjacent Wikipedia paragraphs; in NM (right), full solution traces and DeepSeekR1 reasoning chain.

**Table 1:** Baseline accuracy when models are presented with only the gold context (small or large). Results confirm that all gold variants are independently sufficient to solve the task. Minor fluctuations reflect benchmark variability rather than consistent advantages for larger gold contexts.

	CBB		NQ		NM	
	small	large	small	large	small	large
GPT-4o	0.98	1.00	0.70	0.74	0.93	0.88
Gemini-2.0-Flash	0.96	0.96	0.86	0.80	0.97	0.94
LLaMA-3.1-405b	0.98	1.00	0.81	0.80	0.90	0.92
LLaMA-3.3-70b	0.98	1.00	0.82	0.80	0.89	0.92

**Figure 8:**
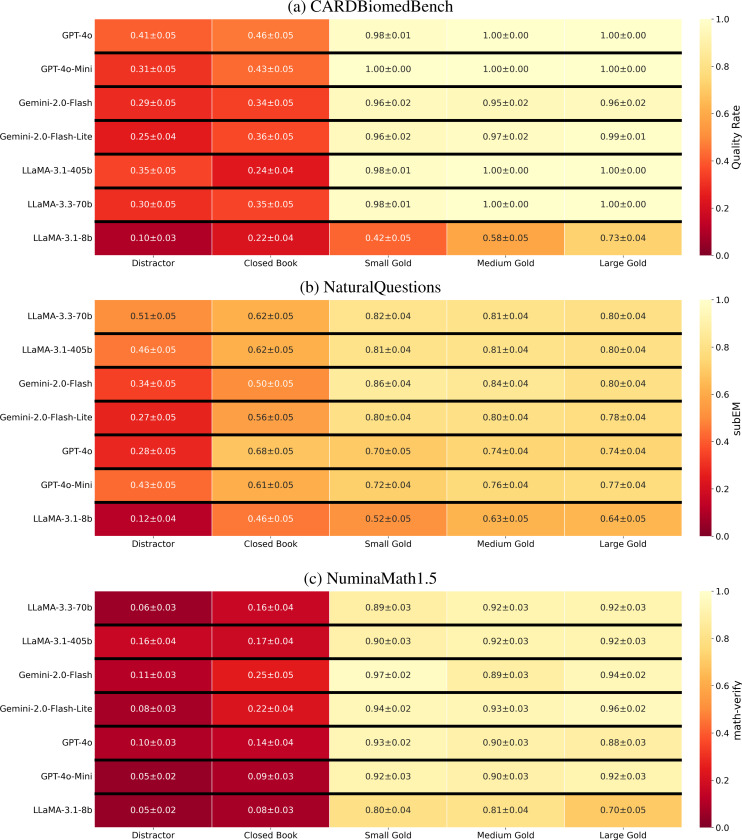
Baseline performance when viewing distractors only, closed book (no documents), and varying sizes of gold. This confirms both (1) models perform poorly without the gold documents and (2) performance is near perfect when viewing any size of gold document.

**Figure 9:**
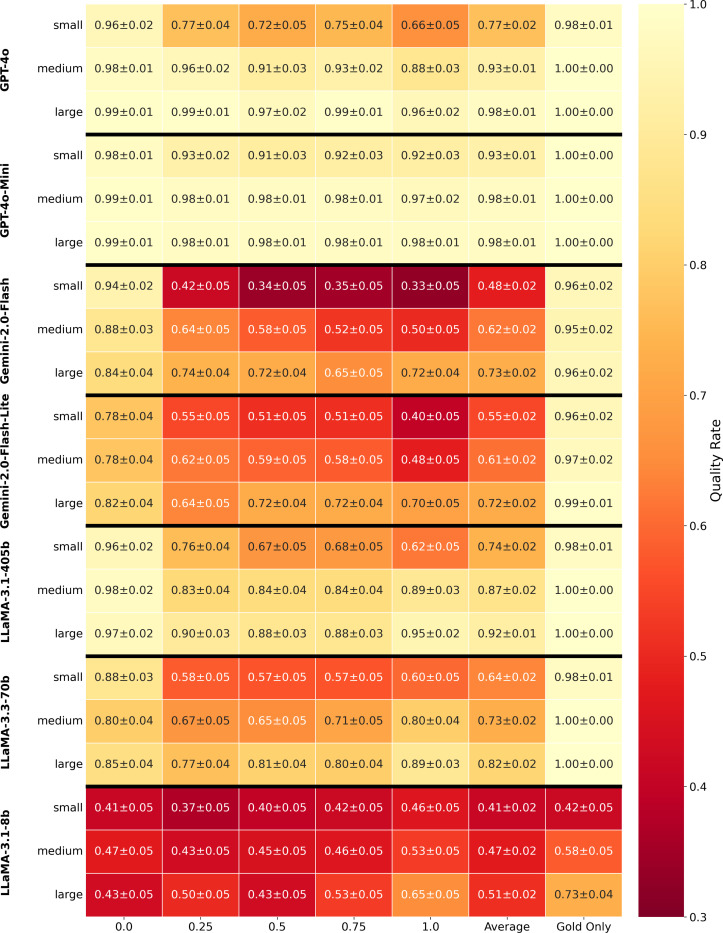
CARDBiomedBench performance for each model and size of gold for varying positions in the context window (0.0, 0.25, 0.5, 0.75, 1.0), the average across all positions, and baseline performance when seeing gold only. Higher scores (light yellow) is more desirable than low scores (dark red), 90% CI are reported.

**Figure 10:**
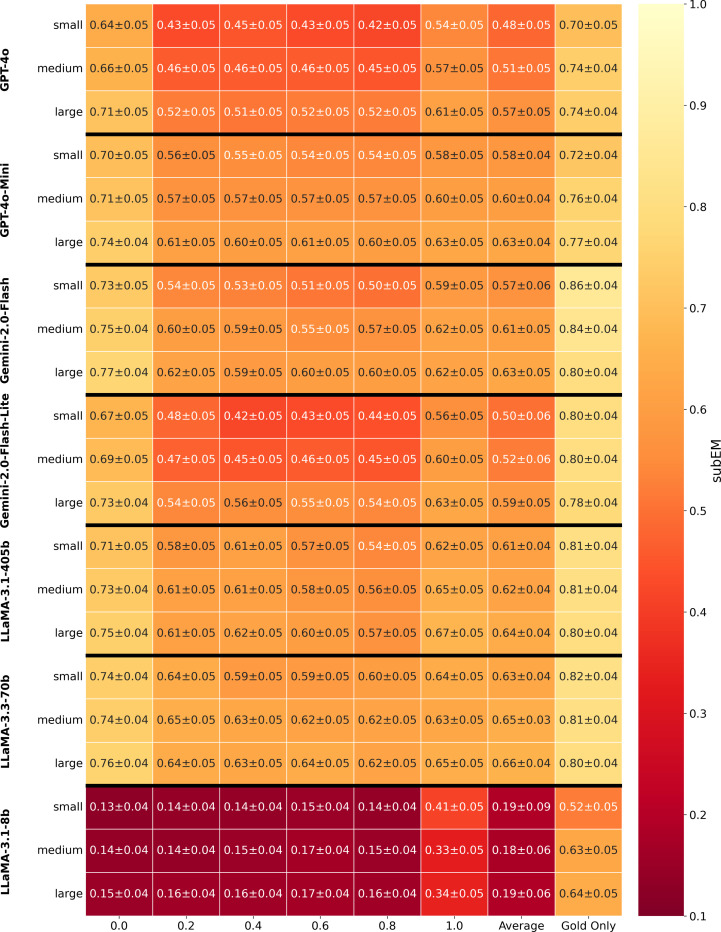
NaturalQuestions performance for each model and size of gold for varying positions in the context window (0.0, 0.2, 0.4, 0.6, 0.8, 1.0), the average across all positions, and baseline performance when seeing gold only. Higher scores (light yellow) are more desirable than low scores (dark red), 90% CI are reported.

**Figure 11:**
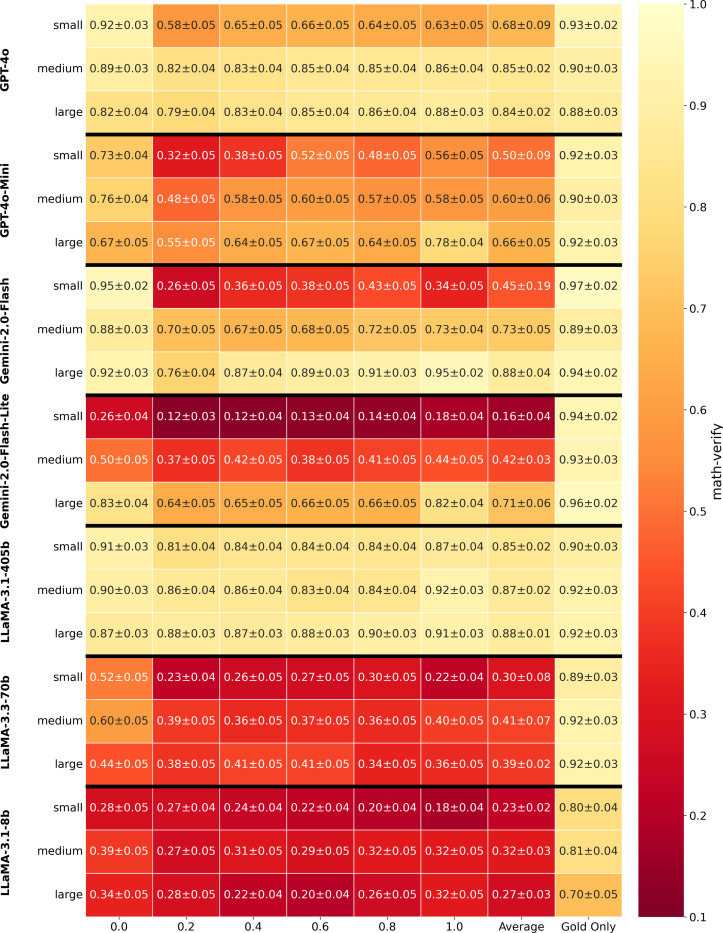
NuminaMath1.5 performance for each model and size of gold for varying positions in the context window (0.0, 0.2, 0.4, 0.6, 0.8, 1.0), the average across all positions, and baseline performance when seeing gold only. Higher scores (light yellow) are more desirable than low scores (dark red), 90% CI are reported.

**Figure 12:**
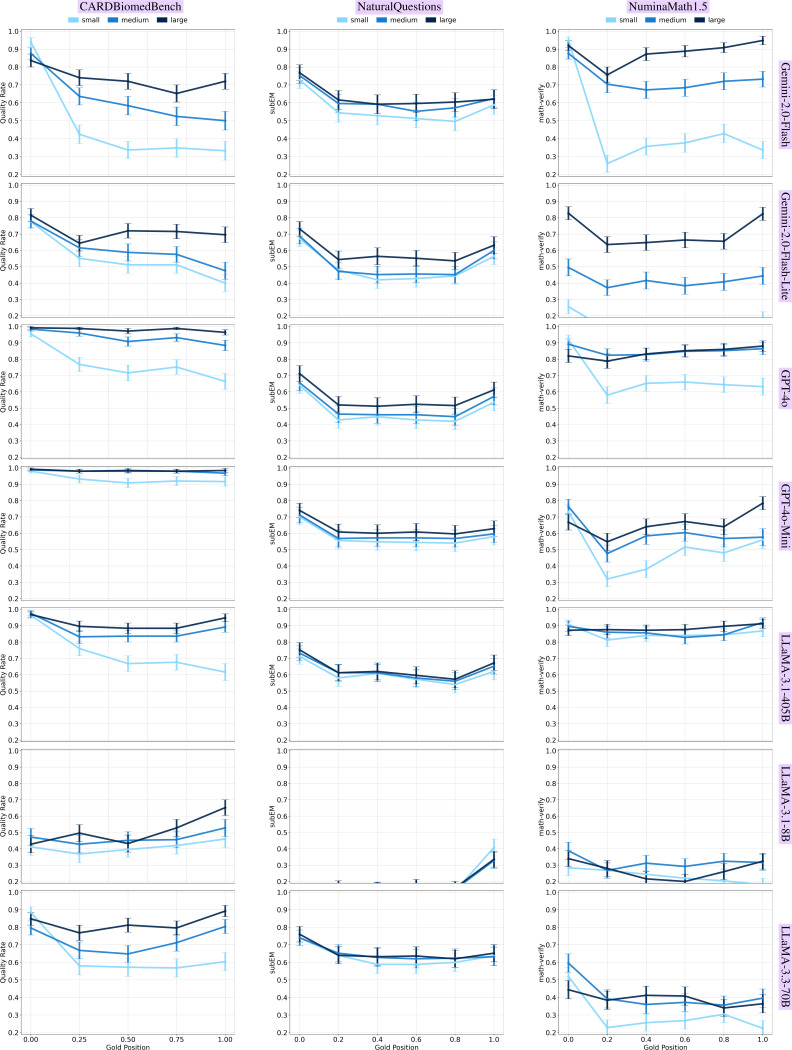
Model performance by gold context position (early to late in input), higher is better and error bars are 90% CIs. Each row is a model, columns are benchmarks. **Smaller gold contexts exhibit sharper performance degradation with later placement, especially in specialized domains (CBB, NM).** Larger contexts mitigate this sensitivity, highlighting the stabilizing effect of richer input. All models, including the ones in Figure 5, are here for comparison.

## A Extended Experimental Details

We provide extended experimental details on benchmark construction, model configuration, and evaluation methodology to support the reproducibility and interpretability of our results.

### A.1 Original Benchmarks

We describe the sources, licenses, and preprocessing procedures for each of the three adapted benchmarks used in our experiments. All experiments were run on a sampled subset of 250 examples per benchmark. See the code repository for exact methodology.

#### CARDBiomedBench

- **Source:** CARDBiomedBench on Hugging Face and its BiomedSQL variant on Hugging Face. Distractor documents were retrieved using a multi-agent retrieval system at NIH, which retrieves content from: (1) Google search over NIH domains, (2) PubTator3.0 [64], (3) the Human Gene Mutation Database (HGMD) [65], and (4) NCBI gene and variant pages [66].
- **License:** Apache 2.0 for benchmark code and data. Some distractor sources (e.g., HGMD) are not redistributable but are publicly accessible on their respective platforms.
- **Preprocessing:** None, the distractor and gold documents are as-is from the retriever.

#### NaturalQuestions

- **Source:** NQ with evidence spans aligned to Knowledge Intensive Language Tasks (KILT) on Hugging Face. Gold documents were loaded using the Ai2 ir_datasets python package [67] and distractors were sourced from HELMET on Hugging Face.
- **License:** Creative Commons Share-Alike 3.0 (NQ), MIT (KILT & HELMET), and Apache 2.0 (ir_datasets).
- **Preprocessing:** We filtered for validation examples that had matching HELMET distractors. Examples with missing KILT provenance, absent or unresolvable answer spans, or malformed metadata were excluded. Gold and distractor documents included the title of the article *‘Title: {title} Document: {gold_document}’* to give them context.

#### NuminaMath1.5

- **Source:** NuminaMath1.5 (NM) and its OpenR1Math (OR1M) variant on Hugging Face, that contains DeepSeekR1 reasoning chains.
- **License:** Apache 2.0. (NM and OR1M).
- **Preprocessing:** Filtered to retain only examples with ‘complete’ and ‘verified’ fields for question, final answer, structured solution, and long-form generation. DeepSeekR1 generations were truncated to the final 5,000 tokens using GPT-4o tiktoken [68] tokenization to normalize document length across tasks. Distractors sampling was among the other questions excluded duplicates. Sizes of gold and distractors were strung into a pseudodocument by including *‘The answer to {question} is {gold_document}’* to give them context.

### A.2 LLM Configuration

We evaluated seven LLMs, each configured via provider-specific APIs. All evaluations were conducted as deterministically as possible.

#### API Providers.

We used the following service providers for model access:

- **GPT models** (GPT-4o, GPT-4o-mini) were accessed via the Azure OpenAI service.
- **Gemini models** (Gemini-2.0-Flash, Gemini-2.0-Flash-Lite) were accessed via the Google AI GenAI SDK, using the official genai Python client.
- **LLaMA models >= 70b params** (Meta-LLaMA-3.1–405B-Instruct, LLaMA-3.3–70BInstruct) were accessed via the Azure AI Inference service.
- **LLaMA model < 70B parameters** (LLaMA-3.1–8B-Instruct) was evaluated locally using the meta-llama/Llama-3.1–8B-Instruct checkpoint, loaded via Hugging Face transformers. All local evaluations were conducted on the NIH High-Performance Computing (HPC) Biowulf cluster [69], leveraging GPU nodes for inference.

#### Prompting and Evaluation Configuration.

Prompts were benchmark-specific and standardized across model types. All models were queried with max_tokens=256 and temperature=0.0. Provider-specific configurations (e.g., safety settings for Google GenAI, and device mapping for HuggingFace) were handled automatically during model initialization. See the code and YAML config files for full details.

#### Grading LLMs.

For CARDBiomedBench, an additional grading LLM was used to assess answer correctness via BioScore using GPT-4o, as done by the authors. It was instantiated using the same infrastructure and configurations as the primary LLMs, with max_tokens=10.

### A.3 Metrics

We used evaluation metrics that align with the original datasets’ scoring protocols:

#### Quality Rate.

We evaluate responses to the CBB tasks following their proposed **BioScore** framework, an LLM-as-a-judge metric implemented with GPT-4o. Each response is scored on a 3-point scale according to the BioScore prompt 4, and a score ≥ 2 is considered factually correct. The **Quality Rate** is computed as the proportion of responses meeting this threshold.

Formally, given a reference set Resp of expert-annotated responses and a corresponding set $R\hat{e}sp$ of model-generated responses for n questions:

(2)
$$\text{Quality Rate}=\frac{1}{N}\sum_{n=1}^{N}Correct\left(r_{n},{\hat{r}}_{n}\right)$$

(3)
$$\text{where}\;Correct\left(r_{n},{\hat{r}}_{n}\right)=\left\{\begin{array}{ll} 1, & \text{if}\;\mathrm{BioScore}\left(r_{n},{\hat{r}}_{n}\right)\geq 2\\ 0, & \text{otherwise} \end{array}\;\text{and}\right.\;r_{n}\in Resp,{\hat{r}}_{n}\in R\hat{e}sp$$

#### SubEM.

For NQ we utilized substring exact match, which assigns a score of 1.0 if any normalized ground truth string is a subspan of the model’s response (after normalization), and 0 otherwise. This is a correctness signal used by previous work on this data.

#### math-verify.

Evaluated with math-verify [70], a symbolic equivalence checker that parses LaTeX boxed answers and verifies correctness through structured math expression comparison. Parsing and verification are done using an extraction and comparison pipeline derived from the Math-Verify toolkit.

#### Error Bars.

All plots showing aggregate scores (e.g., Figure 4) report 90% confidence intervals (CIs) estimated via non-parametric bootstrapping over tasks. Given *N* scores, we resample with replacement 1,000 times and compute the middle 90% interval from the resulting bootstrap distribution.

### A.4 Prompts.

We show prompts used to collect results from the models and the BioScore grading prompt. There is a unique prompt for each benchmark, which is used on every model. {Variables} are in curly braces which are formatted with task data (question and documents). We encourage models to ground their answers in the context and abstain if unable to answer.

You are a highly knowledgeable and experienced expert in the healthcare and biomedical field,
possessing extensive medical knowledge and practical expertise. Create an answer to the question
using only the provided documents (some of which might be irrelevant). If you cannot answer the
question based on the documents, explicitly state that you do not know.
Question: {question}
Documents: {documents}

Prompt 1: The CARDBiomedBench prompt is adapted from the original paper’s experimental methods and includes mention of biomedical expertise.

Create an answer to the question using only the provided documents (some of which might be
irrelevant). If you cannot answer the question based on the documents, explicitly state that
you do not know.
Question : {question}
Documents : {documents}

Prompt 2: The NaturalQuestions prompt is adapted from previous work’s experimental methods [14, 21].

Create an ANSWER to the QUESTION using only the provided DOCUMENTS (some of which might be
irrelevant). Write nothing but your final answer in LaTeX within \\boxed{}. If you do not
know the answer to a question, explicitly state so in \\boxed {I don’t know}.
QUESTION: {question}
DOCUMENTS: {documents}
QUESTION: {question}
ANSWER:

Prompt 3: The NuminaMath1.5 prompt uniquely repeats the question and has formatting guidelines, to comply with the math-verify metric. Without repeating the question models exhibited extremely poor performance in every configuration.

You are a highly knowledgeable and experienced expert in the healthcare and biomedical field, possessing
extensive medical knowledge and practical expertise.
### Scoring Instructions for Evaluating Analyst Responses

**Objective:** Evaluate an analyst’s response against a gold standard.

**Scoring Criteria:**
      - **Exact Match:** 3 points for an exact or equally accurate response.
      - **Close Match:** 2 points for a very close response with minor inaccuracies.
      - **Partial Match:** 1 point for a partially accurate response with significant omissions.
      - **Irrelevant Information (Harmless):** Deduct 0.5 points for harmless irrelevant information.
      - **Irrelevant Information (Distracting):** Deduct 1 point for distracting irrelevant information.
      - **No Match:** 0 points for no match.
      - **Not Knowing Response:** −1 point for stating lack of knowledge or abstaining. An example
      of this scenario is when Analyst Response says \’There are various studies, resources or
      databases on this topic that you can check ... but I do not have enough information on this topic.

**Scoring Process:**
      1. **Maximum Score:** 3 points per question.
      2. **Calculate Score:** Apply criteria to evaluate the response.

**Question:** {question}
**Golden Answer:** {gold_ans}
**Analyst Response:** {pred_ans}

## Your grading
Using the scoring instructions above, grade the Analyst Response return only the numeric score
on a scale from 0.0–3.0. If the response is stating lack of knowledge or abstaining, give it
−1.0.

Prompt 4: BioScore grading prompt for LLM-as-a-judge on CBB tasks, awarding points for correct information and deducting points for incorrect information. It differentiates an abstention (−1) from an incorrect answer (0 or 1).

## B Extended Results

We provide extended baselines for all benchmarks in Figure 8 and main results for CBB in Figure 9, for NQ in Figure 10, and for NM in Figure 11. Additionally, we provide positional curves for all models in Figure 12.

## C Compute Resources

Table 2 details the compute resources needed to reproduce the described experiments. The times listed are approximate execution times (rounded to the nearest 15th minute) from running our experiments but may vary slightly when reproducing results depending on API status and compute resources utilized. CBB experiment runtimes include grading via BioScore with GPT-4o.

**Table 2:** Compute resources needed to reproduce all experiments. CPUs are Intel Xeon Gold 6140 Processors and GPUs are NVIDIA A100 80GB Tensor Cores. Times are listed in terms of Hours:Minutes.

Model	Compute	Memory	CBB Time	NQ Time	NM Time
GPT-4o	2 CPUs	25GB RAM	11:00	03:00	00:30
GPT-4o-Mini	2 CPUs	25GB RAM	08:45	06:15	01:00
Gemini-2.0-Flash	2 CPUs	25GB RAM	06:00	02:30	05:30
Gemini-2.0-Flash-Lite	2 CPUs	25GB RAM	04:30	02:15	00:30
LLaMA-3.1-405b	2 CPUs	25GB RAM	11:45	07:45	02:00
LLaMA-3.3-70b	2 CPUs	25GB RAM	09:15	13:15	13:30
LLaMA-3.1-8b	1 GPU	80GB VRAM	36:30	24:30	06:45
